# PPARs Link Early Life Nutritional Insults to Later Programmed Hypertension and Metabolic Syndrome

**DOI:** 10.3390/ijms17010020

**Published:** 2015-12-24

**Authors:** You-Lin Tain, Chien-Ning Hsu, Julie Y. H. Chan

**Affiliations:** 1 Departments of Pediatrics, Kaohsiung Chang Gung Memorial Hospital, Chang Gung University College of Medicine, Kaohsiung 833, Taiwan; 2 Institute for Translational Research in Biomedicine, Kaohsiung Chang Gung Memorial Hospital, Chang Gung University College of Medicine, Kaohsiung 833, Taiwan; jchan@cgmh.org.tw; 3 Department of Pharmacy, Kaohsiung Chang Gung Memorial Hospital, Kaohsiung 833, Taiwan; cnhsu@cgmh.org.tw; 4 School of Pharmacy, Kaohsiung Medical University, Kaohsiung 807, Taiwan

**Keywords:** developmental programming, hypertension, kidney, metabolic syndrome, nutrient sensing, peroxisome proliferator-activated receptor

## Abstract

Hypertension is an important component of metabolic syndrome. Adulthood hypertension and metabolic syndrome can be programmed in response to nutritional insults in early life. Peroxisome proliferator-activated receptors (PPARs) serve as a nutrient-sensing signaling linking nutritional programming to hypertension and metabolic syndrome. All three members of PPARs, PPARα, PPARβ/δ, and PPARγ, are expressed in the kidney and involved in blood pressure control. This review provides an overview of potential clinical applications of targeting on the PPARs in the kidney to prevent programmed hypertension and metabolic syndrome, with an emphasis on the following areas: mechanistic insights to interpret programmed hypertension; the link between the PPARs, nutritional insults, and programmed hypertension and metabolic syndrome; the impact of PPAR signaling pathway in a maternal high-fructose model; and current experimental studies on early intervention by PPAR modulators to prevent programmed hypertension and metabolic syndrome. Animal studies employing a reprogramming strategy via targeting PPARs to prevent hypertension have demonstrated interesting results. It is critical that the observed effects on developmental reprogramming in animal models are replicated in human studies, to halt the globally-growing epidemic of metabolic syndrome-related diseases.

## 1. Introduction

Metabolic syndrome and its comorbidities may prove to be the greatest crisis to global healthcare. Metabolic syndrome is a cluster of metabolic and cardiovascular symptoms, including insulin resistance, obesity, dyslipidemia, and hypertension. Among these, hypertension is an important component of this syndrome. The origins of susceptibility for many non-communicable diseases in adult, including metabolic syndrome and hypertension, can be traced back to the early life, referred to as “developmental programming” [1]. Early-life nutrition plays an essential role in placental and fetal growth, organogenesis, and development. Given that different nutritional insults can produce the same detrimental consequences that occur in adult life [2,3], a common mechanism might underlie the early-life developmental programming of metabolic syndrome in adulthood [4].

Glucose and related sugars, lipids, and amino acids are important cellular nutrients. The ability to sense and respond to the above environmental nutrient levels is essential for life, which is controlled by nutrient-sensing mechanisms and pathways [5]. Of note is that nutrient sensing nuclear receptors are key integrators of metabolic responses. Peroxisome proliferator-activated receptors (PPARs) are ligand-activated transcription factors of nuclear receptor superfamily involved in the control of nutrition and energy metabolism. To date, three isoforms of PPARs have been identified, namely PPARα, PPARβ/δ, and PPARγ. Importantly, PPARs have been increasingly recognized as key players in the pathogenesis of metabolic syndrome [6]. Although emerging evidence support PPARs may serve as therapeutic targets for treating the metabolic syndrome and its related diseases [7,8], there remains a lack of definitive data on how and when to prevent the developmental programming of hypertension and metabolic syndrome via targeting on PPARs in early life.

Given that the PPARs serve as a nutrient-sensing signaling linking nutritional programming to metabolic syndrome, that the kidney plays a crucial role on the development of the cluster of metabolic diseases [3,9,10], and that PPARs have been implicated in many kidney diseases [8], this review aims to describe the interplay between RRARs, early life nutritional insults, and developmental programming, leading to hypertension and metabolic syndrome in later life, with an emphasis on the kidney.

## 2. The Impact of Renal Programming in Programmed Hypertension and Metabolic Syndrome

A number of mechanisms have been proposed to interpret the programmed hypertension and metabolic syndrome, including thrifty phenotype, catch up growth, glucocorticoid effects, epigenetic changes, and oxidative stress [3,10,11]. However, each of the proposed mechanisms examined in different models of developmental programming was unable fully define a common mechanism, which can drive programmed disease processes. Recently we have shown that programmed hypertension developed in the male offspring of rats exposed to a variety of nutritional insults in early life, including caloric restriction [12], maternal diabetes [13], high-fructose intake [14], postnatal high-salt intake [15], and postnatal high-fat diet [16]. Although several organs control blood pressure (BP), the developing kidney is particularly susceptible to the insults of programming. Data from the human patients and experimental models demonstrated some particular mechanisms in the kidney related to programmed hypertension, including reduced nephron numbers, oxidative stress, epigenetic regulation, activation of the renin-angiotensin system (RAS), and sodium transporters [1,2,9,10,11,12,13,14,15,16,17,18,19,20]. Importantly, renal programming has been identified as a driving mechanism of programmed hypertension [2,10,11].

We recently analyzed the differential expressed genes (DEGs) induced by four different maternal insults in the offspring kidney using next-generation RNA sequencing (NGS) technology [2]. Our NGS data indicate that a diverse range of maternal nutritional insults might generate differentially programmed processes despite the same phenotype—programmed hypertension. Importantly, the PPAR signaling pathway was identified as a significantly regulated Kyoto Encyclopedia of Genes and Genomes (KEGG) pathway and shared by the caloric restriction, maternal diabetes, and high-fructose models [2].

PPARs are nuclear transcription factor and nutrient-sensing signaling that regulate metabolic and tissue developmental processes. Studies focusing on the mechanisms of such metabolic programming have implicated PPARs as a candidate gatekeeper pathway of developmental programming [21,22,23], although their relationship with programmed hypertension and metabolic syndrome remains to be established.

## 3. Peroxisome Proliferator-Activated Receptors (PPARs) and the Kidney in Hypertension and Metabolic Syndrome

The three PPAR isoforms have many aspects of shared biology but different tissue distributions and distinct biological effects [6,7,8]. The three PPARs, by acting as lipid sensors, are involved in regulating fatty acid oxidation, insulin sensitivity, and anti-inflammation. PPARα is expressed mainly in the liver, heart, and kidney. PPARα is predominantly involved in fatty acid utilization, ketogenesis, lipoprotein synthesis, and amino acid catabolism. PPARβ/δ is universally expressed in multiple tissues, especially expressed at high levels in skeletal muscle. Whereas PPARβ/δ function is to regulate adipose tissue metabolism, glucose metabolism, and muscle physiology. PPARγ is highly expressed in adipose tissue and acts as a master regulator of adipogenesis. PPARγ has been implicated in almost all features of metabolic syndrome, including obesity, hypertension, dyslipidemia, insulin resistance, and inflammation. The kidney expresses all three types of PPARs [8].

PPARα ligands have been reported to lower BP in experimental models of hypertension [24,25]. PPARβ/δ activation also elicited antihypertensive effects [26]. However, PPARγ is hypertensive or hypotensive remains inconclusive [8,27]. Abundant clinical evidence suggest that administering PPARγ agonists, particularly thiazolidinedione (TZD) and a subgroup of angiotensin type 1-receptor blockers (ARBs), may be beneficial for patients with hypertension [25,27]. Nevertheless, the BP-lowering effects may be off-target or PPARγ independent [27]. Genetic studies showed the global PPARγ knockout model displays a hypotensive phenotype [28], which is consistent with a report showing that PPARγ stimulates renin gene expression [29]. Since the RAS cascade starts with the release of renin from the kidney, these observations point to a hypertensive role for PPARγ in BP control.

The roles of PPARs in metabolic syndrome has been reviewed and is covered elsewhere [6,7,8]. As the PPARs are involved fundamentally in regulating nutrition and energy homeostasis, they have been considered attractive drug targets for treating metabolic syndrome [7,8]. Among them, PPARγ is the leading therapeutic target and several PPARγ-targeting drugs, such as TZD, has been developed and used clinically [30]. However, little attention has been paid to elucidate whether early target on PPARs can prevent the later development of programmed metabolic syndrome and hypertension.

## 4. PPARs Link Maternal Nutritional Insults to Programmed Hypertension and Metabolic Syndrome

PPAR forms a heterodimer with retinoid X receptor (RXR), which binds to the peroxisome proliferation response element (PPRE), in the promoter region of PPAR target genes. The actions of PPAR are mainly in a ligand-dependent manner. In the absence of ligand, the PPAR/RXR heterodimer recruits co-repressors that maintain PPAR target genes in an inactive state. The addition of ligand causes dissociation of the co-repressors followed by the recruitment of co-activators, such as PPARγ coactivator-1α (PGC-1α) and histone acetyltransferase (HAT), thus inducing the expression of PPAR target genes. Diverse endogenous and synthetic ligands for PPARs have been identified [31], including a variety of nutrients or their metabolites.

Nutrient-sensing signaling pathways reconcile fetal metabolism and development in response to maternal nutritional insults. Accordingly, a number of these signaling pathways exist in the kidney include PPARs, PGC-1α, silent information regulator transcript (SIRT), cyclic adenosine monophosphate-activated protein kinase (AMPK), and mammalian target of rapamycin (mTOR) pathway. SIRT1 and AMPK can mediate deacetylation and phosphorylation of PGC-1α respectively [32], to regulate the expression of PPAR target genes. Next, mTOR has been reported to mediate PPAR activation [33]. Thus, the interplay of PPARs with other nutrient sensing signals allows the kidney to alter the gene expression, morphology, and function in response to maternal nutritional insults and thereby go through a process namely renal programming.

**Figure 1:**
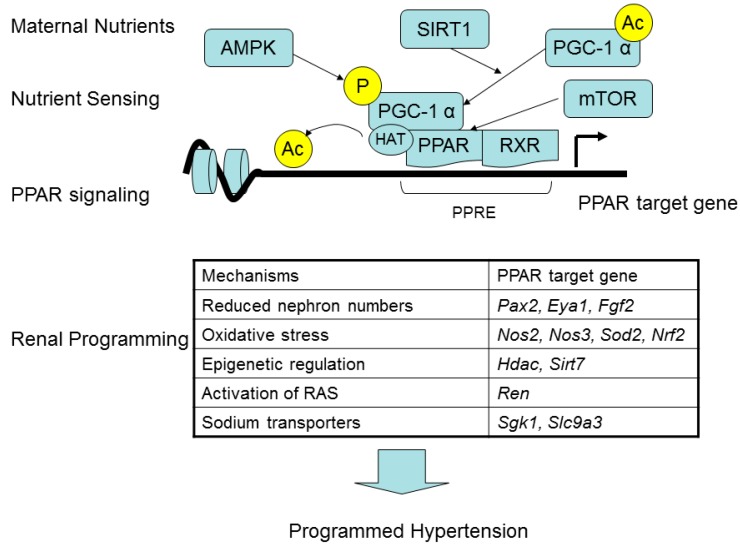
A schema showing the link between maternal nutritional insults and programmed hypertension via PPAR signaling pathway. P, phosphorylation; Ac, acetylation.

So far, there is a number of genes with PPREs or showing primary response to PPARs and their ligands, the so-called PPAR target genes [34,35]. Four lines of evidence suggest that maternal nutritional insults could mediate nutrient sensing mechanisms to regulate PPAR target genes, contributing to programmed hypertension. First, reduced nephron numbers play a role in programmed hypertension [10,11]. Several genes involved in kidney development are PPAR target genes, such as *Pax2* [34], *Eya1* [34], and *Fgf2* [36]. Our previous NGS data demonstrated that *Fgf2* was significantly modified above the chosen threshold in the kidneys of offspring at two weeks of age in response to maternal caloric restriction as well as diabetes [2]. Second, are emerging evidence supports that oxidative stress due to nitric oxide (NO)-reactive oxygen species (ROS) imbalance is important for programmed hypertension [37,38]. A previous report showed that PPARγ can directly regulate a vast array of genes to mediate oxidative stress, including *Nos2*, *Nos3*, *Sod2*, and *Nrf2* [39]. Third, are observations that several PPAR target genes are epigenetic regulators, such as histone deacetylase 5 (*Hdac5*) and *Sirt7* [34,40]. Fourth, are studies showing that several PPAR target genes are belonging to the RAS components or sodium transporters. PPARγ has been reported to stimulate renin gene expression [29]. Next, PPARγ can stimulate serine glucocorticoid kinase-1 (SGK1 encoding *Sgk1* gene) and sodium hydrogen exchanger-3 (NHE3 encoding *Slc9a3* gene) [41]. Taking into considerations that SGK1 can up-regulate several sodium transporters [42] and that increased sodium transporter expression is associated with programmed hypertension [10,11], the alterations in sodium transporters in programmed hypertension is possible a PPAR signaling related mechanism. Therefore, maternal nutritional insults could affect nutrient sensing pathways, especially via PPAR target genes, to induce renal programming leading to programmed hypertension. These heuristic concepts are illustrated in Figure 1.

## 5. PPAR Signaling Pathway in Response to Maternal High-Fructose Intake

Over the past few decades, a rise in metabolic syndrome has been linked to an increase in fructose consumption [43]. Thus, fructose-fed rat, which displays numerous features of the metabolic syndrome, has been generally used as an animal model to study metabolic syndrome and related diseases. Using a maternal high-fructose rat model, we recently found that maternal high-fructose intake induced several phenotypes of metabolic syndrome in adult offspring, including hypertension [14,44]. We used DAVID v6.7 (NIH, Bethesda, MD, USA) to gain biological insight from our NGS dataset [45]. We observed that PPAR signaling pathway is a significant KEGG pathway shared by one-day, three-week, and three-month-old offspring kidney exposed to maternal high-fructose intake [44]. Another significant KEGG pathway shared by three different developmental stages is arachidonic acid metabolism. In this regard, another of our study showing that the protein level and activity of soluble epoxide hydrolase (sEH encoding *Ephx2* gene) are induced by maternal high-fructose exposure in offspring at three months of age [14]. Given that arachidonic acids are ligands for PPARs [7], that the *Ephx2* is a PPAR target gene [35], and that increased expression/activity of sEH have been associated with hypertension [46], these observations implicate a role of PPAR signaling pathway for high-fructose-induced programmed hypertension.

In addition to the kidney, we analyzed DEGs induced by maternal high-fructose intake in the brain stem, liver, skeletal muscle, heart, and urinary bladder in male offspring at one day of age. The chosen criteria of DEGs is (1) minimum of 1.5-fold difference in normalized read counts between groups; and (2) genes that changed by reads per kilo base per million mapped reads (RPKM) ≥0.3 in either control or high-fructose group. As shown in Table 1, we found PPAR signaling pathway is significantly regulated in the liver, heart, and kidney. There were 9, 14, and 19 DEGs related to PPAR signaling pathway identified in the liver, heart, and kidney respectively. Among them, two DEGs, *Fabp4* and *Cd36*, were identified shared by three different organs at postnatal one day. *Fabp4* encodes fatty acid-binding protein 4, is involved in the regulation of glucose and lipid metabolism in relation to inflammatory and metabolic diseases. It has been considered as a biomarker of metabolic and cardiovascular diseases [47]. *Cd36* encodes a class B scavenger receptor CD36 in mediating the inflammation, insulin resistance, and oxidative stress involved in hyperlipidemic states. In the kidney, activation of CD36 and sodium transporter Na/K-ATPase-α1 could form a pro-inflammatory signaling loop to induce hypertension and kidney disease [48].

**Table 1:** Significantly regulated peroxisome proliferator-activated receptor (PPAR) pathway in different organs of maternal high-fructose treated offspring at one day of age.

Organ	Count	Gene Symbol	*p-*Value	Benjamini
Liver	9	*Adipoq*, *Ehhadh*, *Fabp3*, *Fabp4*, *Pparg*, *Cd36*, *Scd1*, *Scl27a5* and *Sorbs1*	5.1 × 10^−2^	4.0 × 10^−1^
Heart	14	*Hmgcs2*, *Acsl1*, *Angptl4*, *Aqp7*, *Cpt1a*, *Cpt1b*, *Ctp2*, *Dbi*, *Fabp4*, *Olr1*, *Rxrg*, *Acaa1*, *Cd36* and *Ubc*	1.1 × 10^−2^	2.1 × 10^−1^
Kidney	19	*Hmgcs2*, *Acsl3*, *Adipoq*, *Angptl4*, *Cpt1b*, *Cyp4a8*, *Cyp4a1*, *Fabp1*, *Fabp4*, *Fabp7*, *Fads2*, *Lpl*, *Me1*, *Ppara*, *Rxrg*, *Acaa1*, *Cd36*, *Ubc* and *Scd*	6.7 × 10^−4^	4.0 × 10^−2^

**Figure 2:**
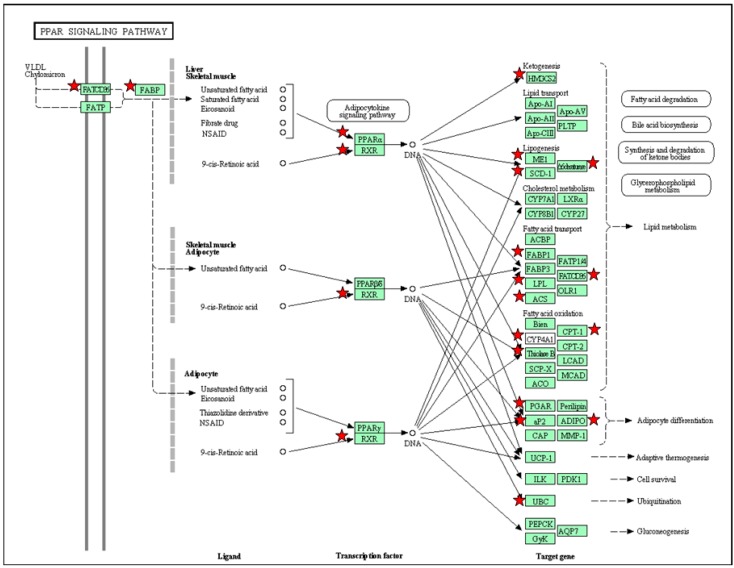
Gene members (green rectangle) in the PPAR signaling pathway that are regulated by maternal high fructose in the male offspring kidney at one day of age (red stars). Data were analyzed using the KEGG pathway feature of the DAVID software [45]. Solid line, downstream signal and PPAR target genes; Dotted line, PPARs regulated mechanisms.

The DEGs in the PPAR signaling pathway in one-day-old offspring kidney in response to maternal high-fructose intake are illustrated in the Figure 2. Most of DEGs in PPAR signaling pathway in the kidney are different from the other organs, suggesting windows of developmental vulnerability to the same insult may be tissue specific. However, all three PPARs and their downstream signaling are more or less involved in the renal programming. All of these findings suggest that PPARs are involved in maternal high-fructose induced programming in a variety of organs and PPAR pathway might be a universally-therapeutic target for programmed hypertension and metabolic syndrome.

## 6. Targeting on PPARs to Prevent Programmed Hypertension and Metabolic Syndrome

There is a vast body of evidence that has indicated that all three PPARs are involved in the pathogenesis of metabolic syndrome and its comorbidities, and their ligands may have therapeutic potential in treating this cluster of metabolic syndrome [6,7,8]. To date, however, only a few studies explored the impact of early intervention by PPAR modulators to prevent programmed metabolic syndrome and its related diseases in programming models, especially nutritional programming-induced acquired hypertension. It is quite conceivable that besides hypertension, many other phenotypes of metabolic syndrome could be influenced by such interventions. Thus, for the sake of brevity, we have restricted this review solely to hypertension. We provide an overview of the different animal models used to achieve reprogramming via PPARs in both genetic and acquired animal models of hypertension.

Natural and synthetic agonists of different PPAR isoforms have been studied in genetic or acquired animal models of hypertension [26,49,50,51,52,53,54,55,56,57,58,59,60,61]. The overview of studies in Table 2 illustrates that the most commonly used reprogramming targets of PPARs are PPARγ agonists. A subgroup of ARBs can act as a partial agonist of PPARγ, which are protective in both genetic and acquired animal models of hypertension. [50,52,59]. Yet their BP-lowering effects may be off-target or PPARγ-independent. On the other hand, selective PPARγ agonists, rosiglitazone and pioglitazone, can be protective in both spontaneously hypertensive rats (SHRs) and developmentally-programmed hypertension [51,56,57]. This may indicate PPAR signaling is a common pathway, whereby both SHRs and acquired programmed hypertension model converge into the same phenotype. However, PPARγ agonists failed to confer antihypertensive effects in the genetic hypertension models of salt-loaded spontaneously-hypertensive stroke-prone rats (SHRSP) [60] and fawn-hooded hypertensive rats (FHH) [61]. It is likely that the extent therapeutic outcomes vary depends on the genetics, types of nutritional insults, timing of exposure, and PPAR agonists utilized. Next, little information is available about the reprogramming effect of PPARβ/δ on programmed hypertension [26], especially in nutritional programming-induced acquired hypertension. Moreover, it is of note that some natural PPAR agonists have been examined in developmentally-programmed hypertension [49,53]. Given fatty acid derivatives with a wide range of affinity to PPARs, it has been difficult thus far to thoroughly evaluate the specificity of each endogenous ligands to the biology of PPARs [31]. Therefore, there remains a long road ahead to determine how and when to apply reprogramming strategies and decide which isoforms of PPARs are an ideal target in a broad range of programmed hypertension models.

**Table 2:** Reprogramming aimed at PPARs in developmentally-acquired and genetic hypertension models.

Programming Model [Reference]	Strain	PPAR Isoform	Treatment	Reprograming Effects
Prenatal dexamethasone exposure [49]	Wistar	PPARα PPARγ	Diet high in ω-3 fatty acids from three weeks to six months of age	Prevented hypertension and hyperleptinemia at six months of age
Low protein diet [50]	Wistar	PPARγ	Losartan between two and four weeks of age	Prevented hypertension at 12 weeks of age
Low protein diet [51]	Wistar	PPARγ	Rosiglitazone from three to six months of age	Prevented hypertension at six months of age
50% caloric restriction [52]	Sprague-Dawley	PPARγ	Losartan between two and four weeks of age	Prevented hypertension at 12 weeks of age
High fat diet [53]	Sprague-Dawley	PPARγ	Conjugated linoleic acid during pregnancy and lactation	Failed to confer antihypertensive effect at 130 days of age
Genetic hypertension [54]	SHR	PPARα	Clofibrate between nine and 12 weeks of age	Prevented hypertension at 12 weeks of age
Genetic hypertension plus high-fat diet [55]	SHR	PPARα	Fenofibrate between 8 and 20 weeks of age	Prevented hypertension at 20 weeks of age
Genetic hypertension [56]	SHR	PPARα PPARγ	Wy14643 or rosiglitazone between five and 13 weeks of age	Prevented hypertension at 13 weeks of age
Genetic hypertension [26]	SHR	PPARβ/δ	GW0742 between 12 and 17 weeks of age	Prevented hypertension at 17 weeks of age
Genetic hypertension [57]	SHR	PPARγ	Pioglitazone between five and seven weeks of age	Prevented hypertension at seven weeks of age
Genetic hypertension [58]	SHR	PPARγ	Magnolol between four and seven weeks of age	Prevented hypertension at seven weeks of age
Genetic hypertension plus high-fat diet [59]	SHR	PPARγ	Telmisartan between eight and 17 weeks of age	Prevented hypertension and renal injury at 17 weeks of age
Genetic hypertension [60]	SHRSP	PPARα PPARγ	Fenofibrate, clofibrate, or rosiglitazone between five and 10 weeks of age	Failed to confer antihypertensive effect at 14 weeks of age
Genetic hypertension [61]	FHH	PPARγ	Pioglitazone from two weeks before birth to four weeks of age	Failed to confer antihypertensive effect at 28 weeks of age

FHH = Fawn-hooded hypertensive rats; SHR = Spontaneous hypertensive rats; SHRSP = salt-loaded spontaneously hypertensive stroke-prone rats.

## 7. Conclusions

Metabolic syndrome in adult life can be programmed by maternal nutritional insults in early life. This concept opens new window to prevent or delay the onset of various phenotypes of metabolic syndrome, like hypertension. The three PPARs, by acting as nutrient sensing signals, are major metabolic regulators and, together, they control BP homeostasis. So far, emerging evidence has indicated that all three PPAR isoforms are involved in the pathogenesis of metabolic syndrome, and their ligands are considered as potential treatments for metabolic syndrome and its related diseases. Studies in short-lived animals, with controlled interventions across their entire lifespan, have provided interesting results on reprogramming strategies to prevent hypertension via targeting on PPARs. However, there remain little data available to decide the “right” PPAR ligand at the “right” time to de-program metabolic syndrome-related hypertension. This is far more challenging in human studies. Although the three PPARs share a large overlap of target gene profile, their resultant phenotypes are quite different in a tissue specific manner. Currently available PPARγ agonists and dual agonists presented unwanted and serious side effects [30,62]. Further efforts are required to develop selective PPAR modulators (SPPARMs) with pharmacological efficiency and minimal adverse effects for patients with metabolic syndrome and associated hypertension. Thereby, a greater understanding of the similarity and difference of three PPARs among a variety of programming models with metabolic syndrome and hypertension is essential to developing early intervention to halt the globally growing epidemic of metabolic syndrome-related diseases.
